# An HPLC-Based Multi-Analyte Secretome Characterization Panel for Canine Adipose-Derived Mesenchymal/Stromal Stem Cells: Quantification of Adenosine, Kynurenine, IL-10, and TGF-β in Conditioned Media—A Pilot Feasibility Study

**DOI:** 10.3390/ijms27093791

**Published:** 2026-04-24

**Authors:** Steven Garner, Emily Laughrun, Susan Mooney, Michael McCord, Seymone Batiste, Melinda Wharton, Rosa Bañuelos, Lori McCord

**Affiliations:** 1 Safari Stem Cell Inc., League City, TX 77573, USA; efoster@safarivet.com (E.L.); mwharton@safarivet.com (M.W.); 2 Safari Veterinary Care Centers, League City, TX 77573, USA

**Keywords:** mesenchymal stromal/stem cells, canine MSCs, secretome characterization, HPLC, adenosine, kynurenine, IL-10, TGF-β, inflammatory priming, conditioned media, immune-mediated hemolytic anemia, IMHA

## Abstract

Mesenchymal stromal/stem cells (MSCs) are increasingly explored for immune-mediated diseases, yet standardized analytical readouts that capture coordinated immunomodulatory output across complementary secretory pathways remain limited. Here, we report the feasibility of an HPLC-based multi-analyte secretome characterization panel that quantifies two small-molecule outputs—adenosine and kynurenine—alongside two immunomodulatory proteins—interleukin-10 (IL-10) and transforming growth factor-beta (TGF-β)—in conditioned media from canine adipose-derived MSCs (cAD-MSCs). Canine immune-mediated hemolytic anemia (IMHA) was used as a disease context to motivate the selection of these analytes, given the pro-inflammatory cytokine environment characteristic of this condition. Three independent cAD-MSC lines were evaluated under baseline conditions and following cytokine stimulation with recombinant interferon-gamma (IFN-γ; 100 ng/mL) and tumor necrosis factor-alpha (TNF-α; 50 ng/mL), referred to herein as inflammatory priming or licensing. Conditioned media were collected at 72 h for metabolite analysis and 48 h for protein analysis, and quantified by HPLC using external calibration and peak integration. Across all three lines, licensing produced directionally consistent increases: mean adenosine increased 2.3-fold, mean kynurenine increased 3.1-fold, mean IL-10 increased 1.6-fold, and mean TGF-β increased 1.7-fold compared with unlicensed controls. Metabolite measurements for adenosine and kynurenine are reported with full chromatographic selectivity data; IL-10 and TGF-β measurements by reversed-phase HPLC with UV detection are presented as exploratory/semi-quantitative outputs and will require orthogonal confirmation (e.g., immunoassay) in future work. These findings are preliminary, derived from three independent donor lines with no comparator group, and are intended to support feasibility of the analytical framework rather than establish definitive performance specifications. Collectively, the data support the potential of a multi-analyte HPLC-based characterization panel to capture licensing-responsive secretory shifts across mechanistically complementary pathways, providing a foundation for expanded development and validation.

## 1. Introduction

Mesenchymal stromal/stem cells (MSCs) are multipotent cells identified in multiple tissue sources, including bone marrow and adipose tissue, and are defined by adherent growth, a characteristic surface marker phenotype (CD73+ CD90+ CD45− CD34−), and the capacity to differentiate along mesenchymal lineages under defined in vitro conditions [1,2]. While early translational interest in MSCs centered on their differentiation potential in orthopaedic models, more recent clinical and preclinical work has increasingly focused on their immune-regulatory properties, which are increasingly recognized as a primary driver of therapeutic activity across a range of inflammatory and immune-mediated conditions [1,3].

In inflammatory disease settings, MSCs reduce immune cell activation and reshape the immune microenvironment through two broad classes of mechanisms: direct cell contact-dependent interactions, including membrane ligand engagement and gap junction communication with T cells and antigen-presenting cells, and the secretion of soluble mediators that act on immune cell populations at a distance [3]. The secreted compartment includes both small-molecule metabolites generated enzymatically and protein mediators produced in response to inflammatory cues. Because these two classes of output operate through distinct molecular pathways, capture of both in a single analytical framework provides a more integrated view of MSC immune-regulatory activity than measurement of either class alone.

A persistent translational challenge is that MSC immune-regulatory output is context-dependent and varies with tissue source, donor biology, culture conditions, and the inflammatory environment [4]. Potency assessment across the field remains heterogeneous, spanning cell-based immune suppression assays, single-pathway surrogate readouts, and secreted-factor measurements, with no universally accepted approach [4,5,6]. Because preferred potency markers and dominant mechanisms can diverge across species, tissue sources, and disease contexts, there is broad recognition that multi-analyte readouts capable of capturing coordinated immune-regulatory output are preferable to single-analyte surrogates [5,6].

To elicit and standardize immune-regulatory output for in vitro testing, inflammatory priming—commonly referred to as licensing—is widely employed. Licensing involves exposure of MSCs to defined pro-inflammatory cytokines prior to or during conditioned media collection, with the goal of driving measurable shifts in secretory output that can be used to evaluate immune-regulatory capacity. Interferon-gamma (IFN-γ) and tumor necrosis factor-alpha (TNF-α) are the most commonly used licensing cytokines in both preclinical and manufacturing contexts; they activate overlapping and complementary intracellular signaling programs in MSCs that upregulate or induce immunomodulatory mediators [7,8]. IFN-γ concentrations of 50–200 ng/mL and TNF-α concentrations of 10–100 ng/mL are routinely used in published MSC licensing studies [7,8,9,10], and concentrations within this range—IFN-γ 100 ng/mL and TNF-α 50 ng/mL—were applied in the present study to provide a defined and reproducible stimulation condition [11].

A well-characterized mechanistic axis activated by IFN-γ licensing is indoleamine 2,3-dioxygenase (IDO), the rate-limiting enzyme in the kynurenine pathway of tryptophan catabolism. IDO catalyzes the oxidative cleavage of the indole ring of L-tryptophan to produce N-formylkynurenine, which is then rapidly hydrolyzed to L-kynurenine (Tryptophan → N-formylkynurenine → Kynurenine) [12,13]. Accumulation of kynurenine in the extracellular environment leads to T-cell suppression and induction of regulatory T-cell (Treg) phenotypes, providing a pathway-level link between inflammatory priming and functional immune modulation [12]. IFN-γ-mediated upregulation of IDO has been described in human and murine MSCs, and IDO activity is frequently used as a potency surrogate in licensed MSC products; canine-specific IDO data are more limited, though the enzymatic pathway is conserved.

MSCs also utilize the adenosinergic pathway to suppress inflammation by converting extracellular adenosine triphosphate (ATP)—present at elevated concentrations in sites of tissue injury and immune activation—into adenosine. This conversion is mediated by the ectonucleotidases CD39 (NTPDase1) and CD73 (5′-nucleotidase), which are expressed on the MSC surface and on MSC-derived extracellular vesicles [14,15]. Extracellular adenosine acts through adenosine receptors on T cells and other immune populations to suppress effector responses and promote Treg differentiation, providing rapid immune dampening in inflammatory environments [14,15]. Because the adenosinergic pathway operates through a distinct mechanism from IDO/kynurenine, pairing adenosine and kynurenine as metabolite readouts provides mechanistically complementary information.

In response to pro-inflammatory signals, MSCs also secrete interleukin-10 (IL-10), an anti-inflammatory cytokine that binds to IL-10 receptor complexes on macrophages and dendritic cells to activate the JAK/STAT3 signaling pathway, thereby inducing anti-inflammatory gene expression and suppressing production of pro-inflammatory mediators including TNF-α, IL-1β, and IL-6 [1,16]. IL-10 also reduces the capacity of antigen-presenting cells to activate CD4+ effector T cells [17]. These effects are primarily mediated through the secreted compartment, though receptor-mediated cell contact can modulate IL-10 responses in co-culture settings.

**Figure 1:**
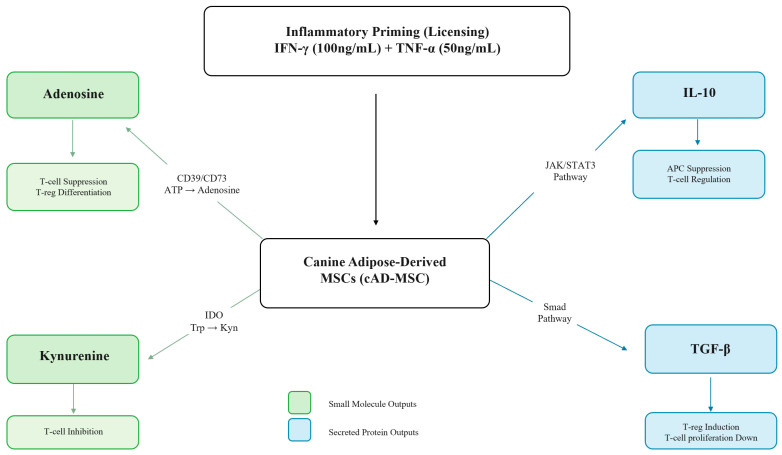
Schematic overview of the four immunomodulatory secretory pathways quantified in this panel. The canine adipose-derived MSC (cAD-MSC) is shown at the center, with upstream inflammatory priming inputs (IFN-γ, 100 ng/mL; TNF-α, 50 ng/mL) activating four coordinated output arms: (1) adenosinergic pathway—ectonucleotidases CD39 and CD73 convert extracellular ATP to adenosine, driving T-cell suppression and Treg differentiation; (2) IDO/kynurenine pathway—IDO-mediated tryptophan catabolism (Tryptophan → N-formylkynurenine → Kynurenine) induces T-cell inhibition and immune tolerance; (3) IL-10—activates JAK/STAT3 signaling to suppress antigen-presenting cell activity and pro-inflammatory cytokine production; (4) TGF-β—signals through the Smad pathway to inhibit T-cell proliferation and induce FoxP3^+^ Treg differentiation. Teal boxes indicate small-molecule metabolite outputs; coral boxes indicate secreted protein outputs. Mechanistic evidence for the adenosinergic and IDO/kynurenine pathways is primarily derived from human and murine MSC studies; direct canine characterization data are limited.

Transforming growth factor-beta (TGF-β) is a pleiotropic cytokine produced by MSCs via Smad-dependent signaling that contributes to longer-term immune suppression. TGF-β suppresses T-cell proliferation through cyclin-dependent kinase inhibitors and reduces IL-2 availability, and also drives induction of FoxP3-expressing Tregs [18,19]. In the context of a secretome characterization panel, TGF-β provides an additional mechanistic dimension by capturing a tolerance-reinforcing output that complements both the rapid metabolite-mediated suppression of the adenosinergic and IDO/kynurenine pathways and the broader anti-inflammatory signaling of IL-10. Figure 1 illustrates the four analyte pathways and their immune-regulatory effects.

For standardized measurement of secreted MSC mediators, immunoassays such as ELISA are widely used because they are sensitive and target-specific. However, reliance on multiple kit-based assays introduces analyte-specific workflows, reagent dependencies, and supply chain considerations that complicate harmonization across manufacturing runs and over time—particularly in veterinary settings where species-validated kits may be limited or require additional cross-reactivity verification [20]. An HPLC-based approach offers an alternative analytical framework in which quantification is anchored to physicochemical separation, external calibration against purified reference standards, and instrument-controlled identity criteria such as retention time and peak integration rules. Although HPLC is most commonly applied to small-molecule analytes, reverse-phase chromatographic methods have been extended to peptide and protein quantification when supported by appropriate sample preparation and reference materials [21,22].

Canine IMHA is used here as a disease context that motivates the biological relevance of the analyte selection. IMHA is characterized by antibody- and complement-mediated erythrocyte destruction with concurrent systemic inflammatory activation, including elevated pro-inflammatory cytokines such as TNF-α [23,24,25,26,27]. The therapeutic rationale for MSC therapy in this setting is to dampen effector immune activity, reduce inflammatory amplification, and support regulatory immune phenotypes that promote tolerance [25,26]. Each of the four analytes quantified in this panel—adenosine, kynurenine, IL-10, and TGF-β—is mechanistically relevant to these goals. A clinical outcomes study using the same allogeneic cAD-MSC platform reported a 76% durable hematologic recovery rate in steroid-refractory IMHA cases [28]; this finding is cited as motivational context for the cell platform and is not presented here as evidence that the secretory profile described in the present study predicts clinical efficacy.

Accordingly, we developed an HPLC-based multi-analyte secretome characterization panel to quantify adenosine, kynurenine, IL-10, and TGF-β in cAD-MSC conditioned media and evaluated its performance across three independent donor lines under baseline and cytokine-licensed conditions. We assessed calibration performance, analyte-specific chromatographic selectivity for the metabolite outputs, and licensing-responsive concentration shifts across all four analytes. The protein analytes (IL-10 and TGF-β) are presented as exploratory/semi-quantitative readouts pending orthogonal confirmation of peak identity. These findings are intended to support feasibility of an HPLC-based multi-analyte characterization framework for MSC secretome assessment, with canine IMHA as a biologically relevant motivating use case.

## 2. Results

### 2.1. HPLC Calibration Performance

**Figure 2:**
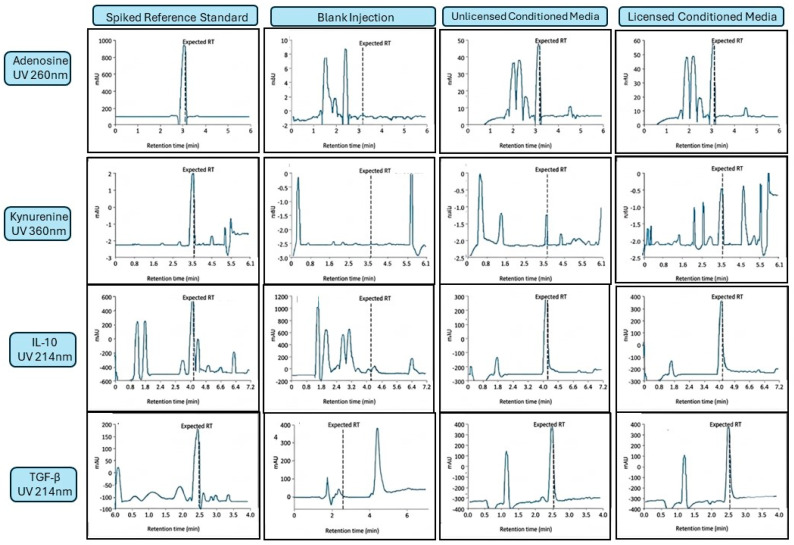
Representative HPLC chromatograms for all four panel analytes under reference standard, blank medium, unlicensed conditioned media (CM), and licensed CM conditions. The dashed vertical line in each panel marks the expected retention time based on co-injection of purified reference standards; detection wavelengths were UV 260 nm (adenosine), UV 360 nm (kynurenine), and UV 214 nm (IL-10 and TGF-β). Earlier-eluting peaks visible in adenosine and kynurenine CM panels represent matrix components retained after sample preparation and are chromatographically resolved from the analyte peak. For IL-10 and TGF-β, peak identity in CM samples was assigned by retention-time matching to purified reference standards and has not been orthogonally confirmed; early-eluting peaks (~1–2 min) in the IL-10 blank medium reflect highly polar matrix components with minimal C18 retention; these are fully resolved from the analyte retention time (~4 min) and do not contribute to quantification. These measurements are therefore exploratory. *Y*-axis scales are set independently per panel. CM, conditioned media; mAU, milliabsorbance units.

Representative HPLC chromatograms for all four panel analytes under reference standard conditions, blank culture medium, unlicensed conditioned medium, and licensed conditioned medium are provided in Figure 2. For adenosine (UV 260 nm) and kynurenine (UV 360 nm), the selectivity of the respective detection wavelengths and sample preparation steps (10 kDa MWCO filtration for adenosine; TCA protein precipitation for kynurenine) substantially reduced protein and macromolecular background. Reference standards of each metabolite resolved as discrete peaks with clean baseline separation from residual matrix components in processed blank medium, and the corresponding peaks in conditioned media chromatograms co-eluted with the respective standards at matching retention times. Spiked recovery experiments performed in a conditioned media matrix confirmed quantitative recovery of adenosine (mean recovery: 100.0% ± 5.5%) and kynurenine (mean recovery: 98.2% ± 3.6%) across the evaluated concentration ranges, supporting acceptable analytical accuracy for both analytes and demonstrated the absence of significant co-eluting interferents at the analyte retention times under unlicensed and licensed conditions.

For IL-10 and TGF-β, quantification was performed by reversed-phase HPLC with UV detection at 214 nm, a wavelength that detects peptide bond absorption non-selectively. Conditioned media were concentrated 20-fold prior to injection (see Section 4.4.3), and peak identity was assigned by retention-time matching to purified reference standards evaluated in blank medium and in concentrated matrix. It is acknowledged that UV 214 nm detection in complex conditioned media cannot definitively establish analyte identity without orthogonal confirmation, and the reported IL-10 and TGF-β concentrations should be interpreted as exploratory/semi-quantitative estimates pending independent verification (e.g., by immunoassay or mass spectrometry). Despite this limitation, the directionally consistent increases observed across all three donor lines following licensing are reported to document panel behavior and inform future analytical development.

### 2.2. HPLC Calibration Performance Across the Panel

External calibration curves demonstrated strong linearity across the working ranges for all four analytes (Table 1). Coefficients of determination (R2) were ≥0.9939 for all analytes, supporting linear back-calculation of concentrations from peak areas under the stated detection channels. The validated LOQ, defined as the lowest concentration producing a signal-to-noise ratio ≥ 10 with back-calculated accuracy within ±15% of nominal, was 0.1 µM for adenosine, 1 µM for kynurenine, and 0.5 ng/mL for IL-10 and TGF-β. For the protein analytes, the LOQ was determined in the concentrated sample (approximately 20-fold pre-concentration from the original conditioned media volume); reported conditioned media concentrations in Table 2 and Table 3 represent values back-calculated to original media equivalents by applying the appropriate concentration factor. One mid-range IL-10 calibration standard (25 ng/mL) back-calculated at 14% deviation from nominal, which falls outside the commonly applied ±10% acceptance criterion; this point is flagged in Table 1 and the IL-10 calibration will be revisited in subsequent method development.

### 2.3. Licensing-Responsive Shifts Across the Secretome Panel

**Figure 3:**
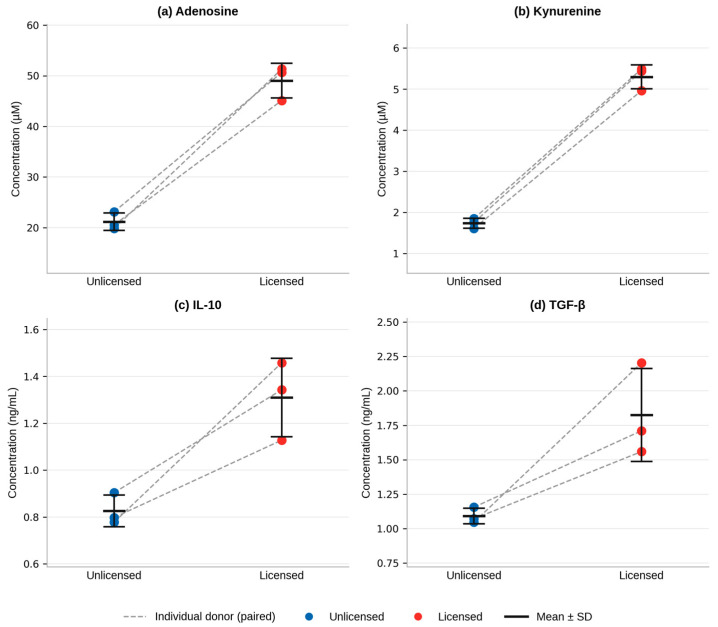
Licensed vs. unlicensed conditioned media outputs quantified by HPLC across the panel. Paired dot plots show individual concentrations for each of the three independent canine adipose-derived MSC donor lines (connected dashed lines indicate paired observations from the same donor) for (**a**) adenosine, (**b**) kynurenine, (**c**) IL-10, and (**d**) TGF-β. Horizontal bars indicate group means; error bars indicate ±1 SD (n = 3). “Licensed” indicates conditioned media collected following cytokine stimulation with IFN-γ (100 ng/mL) + TNF-α (50 ng/mL); “Unlicensed” indicates unstimulated controls. For all analytes: one-tailed paired Wilcoxon signed-rank test, W = 6, r = 1.00, *p* = 0.125 (n = 3; *p* = 0.125 is the minimum achievable value at this sample size). IL-10 and TGF-β measurements are exploratory/semi-quantitative; peak identity was assigned by retention-time matching to reference standards.

Across three independent cAD-MSC lines, cytokine stimulation (licensing) produced higher conditioned-media concentrations for all four panel analytes compared with unstimulated controls (Figure 3; Table 2). This panel-wide response was directionally consistent across all three donor lines for every analyte (Table 3). Given the pilot scope of this study (n = 3), statistical analysis was necessarily exploratory. One-tailed paired Wilcoxon signed-rank tests showed that all three donor lines changed in the licensed direction for every analyte (W = 6, r = 1.00, *p* = 0.125 for each analyte; exact method, n = 3). The rank-biserial correlation of 1.00 reflects the maximum possible effect size, as no donor line showed a decrease in any analyte following licensing. Bootstrap 95% confidence intervals for mean fold-change were entirely above 1.0 for all analytes: adenosine, 2.19–2.59; kynurenine, 2.97–3.11; IL-10, 1.41–1.87; and TGF-β, 1.46–2.11. Kynurenine showed the narrowest interval (range, 0.14), consistent with its low between-line CV of 5.5%, whereas TGF-β showed the widest interval (range, 0.65), consistent with greater between-donor variability (CV 18.5%). Because n = 3 limits statistical power, the *p*-value of 0.125 should not be interpreted as evidence against a true licensing effect; rather, these findings support a consistent preliminary licensing-responsive trend that should be confirmed in a larger donor set.

### 2.4. Analyte-Specific Responses to Licensing

#### 2.4.1. Adenosine

Adenosine increased from 21.180 ± 1.733 µM in unlicensed conditioned media to 49.032 ± 3.413 µM following licensing (2.32-fold increase; Figure 3a; Table 2). Between-line variability was low for both conditions (CV 8.2% unlicensed, 7.0% licensed), and all three donor lines showed increases in the same direction (Table 3). Spiked recovery in conditioned media matrix confirmed chromatographic selectivity at the adenosine retention time (see Section 2.1).

#### 2.4.2. Kynurenine

Kynurenine increased from 1.736 ± 0.121 µM under unlicensed conditions to 5.298 ± 0.292 µM following licensing (3.05-fold; Figure 3b; Table 2). Between-line variability was low for both conditions (CV 6.9% unlicensed, 5.5% licensed). Kynurenine showed the largest proportional response to licensing among the four panel analytes, consistent with robust IDO pathway induction.

#### 2.4.3. IL-10

IL-10 increased from 0.826 ± 0.068 ng/mL in unlicensed conditioned media to 1.309 ± 0.168 ng/mL following licensing (1.58-fold; Figure 3c; Table 2). Between-line variability was low for unlicensed conditions (CV 8.2%) and moderate for licensed conditions (CV 12.8%). As noted in Section 2.1, these measurements are exploratory/semi-quantitative; all reported concentrations fall above the validated LOQ of 0.5 ng/mL and are pending orthogonal identity confirmation.

#### 2.4.4. TGF-β

TGF-β increased from 1.092 ± 0.057 ng/mL under unlicensed conditions to 1.825 ± 0.337 ng/mL with licensing (1.67-fold; Figure 3d; Table 2). Between-line variability was notably higher for licensed TGF-β (CV 18.5%) compared with other analytes, consistent with known biological heterogeneity in TGF-β secretory output across MSC donors. As with IL-10, reported values are exploratory estimates pending orthogonal confirmation.

### 2.5. Line-Level Consistency Across Independent MSC Lines

Individual-line concentrations are provided in Table 3. Directional increases with licensing were observed across all three independent donor lines for every panel analyte, indicating that panel-wide effects were not attributable to a single outlier line. Metabolite outputs (adenosine and kynurenine) showed consistent between-line behavior; protein outputs (IL-10 and TGF-β) showed somewhat higher between-line variation, most notably for licensed TGF-β, consistent with expected biological heterogeneity across donors.

## 3. Discussion

This pilot study assessed the feasibility of an HPLC-based multi-analyte secretome characterization panel capable of quantifying adenosine and kynurenine—two small-molecule immune-regulatory metabolites—alongside IL-10 and TGF-β from cAD-MSC conditioned media. Cytokine licensing with IFN-γ and TNF-α produced directionally consistent, panel-wide increases across all four analytes in all three independent donor lines, supporting the concept that this analytical framework can detect licensing-responsive secretory shifts across mechanistically distinct pathways. The findings are preliminary and are presented with explicit acknowledgment of the small sample size (n = 3), the exploratory status of the protein measurements, and the absence of full analytical validation or functional correlation data.

### 3.1. Interpretation of Licensing-Responsive Metabolite Outputs

Kynurenine increased approximately 3-fold following licensing, the largest proportional response across the panel. This is consistent with well-characterized IDO pathway biology in which IFN-γ upregulates IDO1 expression, driving tryptophan catabolism and kynurenine accumulation [12,13]. The published evidence for IDO-mediated tryptophan degradation as a T-cell suppressive mechanism is primarily derived from human and murine MSC studies [12]; canine-specific IDO characterization data are limited, and direct mechanistic attribution in cAD-MSCs should be approached cautiously. Dose- and duration-dependent effects of IFN-γ on IDO expression and kynurenine output have been documented across MSC preparations [9], supporting the expectation that the specific stimulation conditions used here (100 ng/mL IFN-γ) would produce detectable kynurenine shifts.

Adenosine increased approximately 2.3-fold after licensing. Adenosinergic immunosuppression by MSCs has been linked to CD39/CD73-mediated nucleotide hydrolysis, and its dependence on co-stimulation by immune cells has been noted in human MSC studies [29]. As with kynurenine, extrapolation of specific mechanistic details from human data to the canine system should be made with care, although the ectonucleotidase pathway is enzymatically conserved. Because the adenosinergic and IDO/kynurenine pathways operate through distinct enzymatic mechanisms, their co-measurement in the same panel captures complementary suppressive outputs and avoids the interpretive limits of single-pathway readouts [5,6].

### 3.2. Interpretation of Licensing-Responsive Protein Outputs

IL-10 and TGF-β showed 1.6- and 1.7-fold increases following licensing, respectively. The directionality of these changes is consistent with a broad framework in which licensed MSCs and MSC-immune cell interactions promote anti-inflammatory and tolerance-supporting signaling [30,31]; however, the mechanistic evidence supporting IL-10 and TGF-β secretion by MSCs in response to IFN-γ/TNF-α stimulation derives largely from human and murine MSC literature, and canine-specific protein secretion data are limited. More critically, the HPLC measurements for IL-10 and TGF-β must be interpreted with caution. Reversed-phase HPLC with UV detection at 214 nm is a non-selective detection strategy in complex conditioned media: the sample matrix contains numerous UV-absorbing proteins and peptides that could co-elute with the target analytes. While peak identity was assigned by retention-time matching to purified reference standards run in blank medium and concentrated matrix, this approach alone does not constitute definitive identity confirmation. The observed peaks could, in part, reflect co-eluting matrix components. Future work should include spike-recovery experiments in conditioned media matrix and orthogonal confirmation of peak identity by immunoassay, immunoprecipitation, or mass spectrometry before the protein outputs are used quantitatively.

### 3.3. Value of an Integrated, Single-Platform Panel for Standardization

A key objective of this work was to evaluate whether an instrument-based analytical framework could provide a harmonized readout of mechanistically complementary MSC secretory outputs. Chromatographic quantification anchors measurement to physicochemical separation and external calibration criteria that are independent of species-specific antibody supply chains, which is an advantage in veterinary settings where validated immunoassay kits may be limited [20]. However, the present study also illustrates the boundary of this approach: HPLC is well-suited to discrete small-molecule analytes with defined spectroscopic properties and clean separation from matrix, as demonstrated here for adenosine and kynurenine, but requires additional validation investment to establish specificity for protein analytes in complex biological matrices.

It should be noted that the four analytes were measured under distinct analytical conditions: adenosine and kynurenine were processed with serum-containing conditioned media using C18 columns with analyte-specific mobile phases and detection wavelengths, and IL-10 and TGF-β from 48-h serum-free conditioned media on a wide-pore C18 column at 214 nm. These constitute analyte-specific HPLC methods performed on the same instrument platform, not a single unified workflow; the practical advantage is instrument consolidation rather than fully integrated method harmonization. The different collection timepoints mean that cross-analyte comparisons of fold-change represent responses under different temporal conditions and should not be interpreted as a single secretory snapshot. Justification for and implications of the timepoint differences are addressed in Section 4.2.

### 3.4. Canine IMHA as a Clinically Relevant Use Case for Inflammation-Responsive Output

Canine IMHA is used here as a motivational disease context rather than a primary analytical endpoint. The clinical relevance is grounded in the pro-inflammatory cytokine environment of active IMHA, as dogs with this condition demonstrate elevated circulating TNF-α [23], and the therapeutic rationale for MSC therapy in dampening immune effector activity while supporting tolerance-promoting pathways [25,26]. These features make IMHA a biologically coherent context in which to evaluate licensing-responsive MSC secretory output. The present study contains no patient samples and makes no claims about the relationship between the secretory profile documented here and clinical therapeutic outcomes.

### 3.5. Limitations and Future Directions

This study has several important limitations that should be considered when interpreting the findings. First, the pilot scope (n = 3 independent MSC lines) limits statistical power; the sign test analysis confirms directional consistency but cannot achieve significance at conventional thresholds. Conclusions about panel sensitivity, expected ranges, or between-lot discrimination require expanded donor sets. Second, IL-10 and TGF-β measurements are exploratory and semi-quantitative: peak identity has not been orthogonally confirmed, and the measurements may include contributions from co-eluting matrix components. Third, the panel as currently implemented uses two distinct collection timepoints (72 h for metabolites, 48 h for proteins) and two separate media conditions (serum-containing and serum-free), meaning the four analytes do not represent a contemporaneous secretory snapshot. Fourth, full analytical validation—including selectivity against matrix interferents, spiked recovery, intra- and inter-day precision, robustness, and system suitability criteria—has not been completed; the parameters assessed (linearity, LOQ by signal-to-noise ratio, back-calculated accuracy) represent a starting point rather than a validated method package.

## 4. Materials and Methods

### 4.1. MSC Lines

Three independent canine adipose-derived MSC (cAD-MSC) lines were used. All lines were derived from adipose tissue collected from client-owned female dogs undergoing elective ovariohysterectomy procedures under sedation and general anesthesia; tissue was collected as surgical by-product with informed owner consent. Lines were confirmed as MSCs by flow cytometric immunophenotyping (CD90+ CD73+ CD45− CD34−) and trilineage differentiation capacity (adipogenic, chondrogenic, osteogenic), as detailed in the donor eligibility and characterization procedures described in Garner and Laughrun (2025) [28]. The three cAD-MSC lines used in the present study were derived from a separate donor cohort from those reported in reference [28]; the clinical outcomes data in that publication do not overlap with the donors or samples in the present study.

### 4.2. Cell Culture and Expansion

MSC lines were expanded to passage 5 and maintained in DMEM/F12 supplemented with 10% fetal bovine serum (FBS) and 1% penicillin-streptomycin (Thermo Fisher, Scientific, Waltham, MA, USA). Cells were cultured at 37 °C in a humidified incubator with 5% CO_2_ and plated in 25 cm^2^ flasks. Cytokine stimulation was initiated when cultures reached approximately 70% confluency.

### 4.3. Inflammatory Licensing and Conditioned Media Collection

Cytokine stimulation (licensing) was performed using recombinant IFN-γ (100 ng/mL) and TNF-α (50 ng/mL) (Thermo Fisher, Scientific, Waltham, MA, USA). These concentrations were selected based on published MSC licensing studies in which similar IFN-γ (50–200 ng/mL) and TNF-α (10–100 ng/mL) concentrations are routinely used to activate IDO-mediated and related immune-regulatory programs [7,8,9,10], and to provide a defined stimulation condition within the range that has been shown to produce measurable IDO expression and secretory shifts in MSC preparations. Unlicensed controls were handled in parallel under identical conditions without added cytokines.

For small-molecule analyses (adenosine and kynurenine), conditioned media were collected after 72 h of cytokine exposure in standard DMEM/F12 + 10% FBS, reflecting the timepoint used for metabolite accumulation in published MSC co-culture and conditioned media studies.

For protein analyses (IL-10 and TGF-β), the media was switched to serum-free DMEM/F12 + 1% penicillin-streptomycin for a 48 h collection window, to reduce albumin-associated matrix interference that would otherwise dominate the UV 214 nm chromatogram and complicate protein peak resolution. The different timepoints reflect the distinct analytical constraints of metabolite vs. protein detection and are a recognized limitation: cross-analyte comparisons of fold-change magnitude cannot be interpreted as representing contemporaneous secretory output. Future work will evaluate whether harmonized timepoints and matrix conditions (e.g., serum-free collection for all analytes, or depletion of albumin from serum-containing media) can reduce this constraint. Conditioned media were clarified by centrifugation (16,000× *g*, 5 min) to remove particulates prior to downstream processing.

### 4.4. Conditioned Media Processing and Sample Preparation

All reagents were HPLC grade unless otherwise noted. Media samples were filtered through a pre-wetted 0.22 µm PES membrane filter prior to analyte-specific processing (Millipore Sigma, Burlington, MA, USA).

#### 4.4.1. Adenosine Sample Preparation

Adenosine was measured from the low-molecular-weight filtrate generated by a 10 kDa MWCO centrifugal filter (pre-wetted per manufacturer instructions) (Thermo Fisher, Scientific, Waltham, MA, USA). This step removes macromolecular protein background while retaining small nucleoside analytes. The filtrate was injected directly for HPLC analysis.

#### 4.4.2. Kynurenine Sample Preparation

Kynurenine was prepared by TCA protein precipitation: clarified conditioned media were mixed with trichloroacetic acid (TCA) to a final concentration of 5% (*v*/*v*), incubated on ice for 10 min, and centrifuged at 16,000× *g* for 5 min. The supernatant was diluted 1:1 (*v*/*v*) with the initial mobile phase prior to injection (Thermo Fisher, Scientific, Waltham, MA, USA). This step removes high-molecular-weight proteins while retaining kynurenine and related small metabolites.

#### 4.4.3. IL-10 and TGF-β Sample Preparation

Protein-containing conditioned media (serum-free) were concentrated using a 10 kDa MWCO centrifugal filter (Thermo Fisher, Scientific, Waltham, MA, USA). Approximately 20 mL of conditioned media per condition was concentrated to approximately 1 mL (nominal 20-fold concentration factor). The concentration factor was applied to improve signal-to-noise ratio for low-abundance protein analytes at UV 214 nm. Following concentration, trifluoroacetic acid (TFA) was added to a final concentration of 0.1% (*v*/*v*) prior to injection. All reported conditioned media concentrations were measured directly in the concentrated sample and fall above the LOQ of 0.5 ng/mL; note that concentrating the matrix also concentrates all matrix components proportionally, which is a recognized limitation for peak selectivity. A serum-free blank (DMEM/F12 + 1% penicillin-streptomycin, processed identically through the 20× MWCO concentration step) was injected alongside study samples and confirmed the absence of medium-derived peaks at the IL-10 and TGF-β analyte retention times; large early-eluting peaks observed in concentrated blank medium (~1–1.5 min) and a late-eluting peak (~6 min) represent UV-absorbing medium constituents and are chromatographically resolved from the analyte elution window.

### 4.5. HPLC Instrumentation and Chromatographic Conditions

Analyses were performed on an Agilent 1200 Series HPLC system (Agilent Technologies, Santa Clara, CA, USA). Small-molecule separations (adenosine and kynurenine) were performed on a Thermo Hypersil GOLD C18 column (4.6 × 150 mm, 3 µm particle size; Thermo Fisher, Scientific, Waltham, MA, USA). Protein separations (IL-10 and TGF-β) were performed on a Millipore Discovery Bio Wide Pore C18 column (4.6 × 150 mm, 5 µm particle size; Millipore Sigma, Burlington, MA, USA). The use of two distinct columns and column chemistries reflects the different retention and elution requirements of small-molecule nucleoside/amino-acid analytes vs. larger protein analytes. Processed samples were injected in duplicate, and the mean peak area was used for quantification.

#### 4.5.1. Adenosine Method (UV 260 nm)

Mobile phase A: water + 0.1% TFA (*v*/*v*). Mobile phase B: acetonitrile + 0.1% TFA (*v*/*v*). Isocratic conditions at 5% B, 1.0 mL/min. Column temperature: 35 °C. Adenosine was quantified by UV absorbance at 260 nm, corresponding to the purine ring absorption maximum, which provides high selectivity for nucleoside analytes in the filtrate.

#### 4.5.2. Kynurenine Method (UV 360 nm)

Mobile phase A: 15 mM sodium acetate buffer (pH 4.6). Mobile phase B: acetonitrile + 0.1% TFA (*v*/*v*). Isocratic at 5% B for 7 min, followed by column wash and re-equilibration, 1.0 mL/min. Column temperature: 35 °C. Kynurenine was quantified at 360 nm, a wavelength at which kynurenine exhibits strong absorbance due to its conjugated carbonyl chromophore, providing selectivity relative to other TCA precipitate components.

#### 4.5.3. Protein Method for IL-10 and TGF-β (UV 214 nm)

Mobile phase A: water + 0.1% TFA (*v*/*v*). Mobile phase B: acetonitrile + 0.1% TFA (*v*/*v*), at 0.8 mL/min. Gradient: 2% B hold for 2 min; linear ramp to 40% B over 10 min; wash and re-equilibration for 8 min. Column temperature: 45 °C. UV detection at 214 nm, corresponding to peptide bond absorption. This wavelength detects all peptide-bonded analytes non-selectively.

### 4.6. Peak Identity Confirmation, Calibration, and Quantification

Peak identity was assigned by co-injection of purified reference standards at the target retention time, evaluated in blank culture medium and in concentrated/processed conditioned media matrix. For adenosine and kynurenine, spiked recovery experiments in conditioned media matrix were performed to confirm that matrix components do not co-elute at the analyte retention time and to verify quantitative recovery. For IL-10 and TGF-β, retention-time matching was the sole identity confirmation method used in this study; orthogonal confirmation (spike recovery in concentrated CM matrix, ELISA cross-check, or mass spectrometric verification) is planned for future method development and is required before protein outputs are used quantitatively.

External calibration curves were generated by serial dilution of purified reference standards and fit by linear regression (peak area vs. concentration) (ChemStation Software (version Rev. B.04.03) and Excel). The validated LOQ was defined as the lowest standard concentration yielding a signal-to-noise ratio ≥ 10 and back-calculated accuracy within ±15% of nominal. Back-calculated accuracy was within ±10% of nominal for all adenosine, kynurenine, and IL-10 standards; for IL-10, one mid-range standard showed 14% deviation (outside the conventional ±10% criterion, flagged for future refinement). Where concentration or dilution factors were applied, calculated concentrations were adjusted accordingly.

### 4.7. Analytical Validation Scope

This feasibility study assessed the following analytical performance parameters: calibration linearity (R^2^), LOQ by signal-to-noise ratio criterion, and back-calculated accuracy of calibration standards. The following validation parameters were not evaluated in the present study and remain as planned work for future method development: matrix effects, extraction recovery, intra-day and inter-day precision, robustness, system suitability criteria, and carryover. Reporting of these outstanding parameters is required before the methods can be considered validated for routine regulated use.

### 4.8. Statistical Analysis

This study was designed as a pilot feasibility evaluation using three biologically independent canine adipose-derived MSC lines. For each analyte, conditioned media from licensed and unlicensed cultures were generated separately for each MSC line, and each donor-derived line was treated as one biological replicate. Where multiple HPLC injections were performed for the same processed sample, peak areas were averaged prior to concentration back-calculation and downstream summary, such that reported values represent one concentration value per line per condition.

Data are presented as mean ± standard deviation (SD) across the three independent MSC lines. Coefficient of variation (CV%) was calculated as (SD/mean) × 100. Fold-change was calculated as the ratio of mean licensed concentration to mean unlicensed concentration for each analyte. Reported SD and CV values reflect between-line biological variation for each condition after averaging technical injection replicates.

The limit of quantification (LOQ) for each analyte was defined as the lowest concentration that produced a signal-to-noise ratio ≥ 10 and acceptable back-calculated accuracy within the calibration model. Signal-to-noise ratios were determined from chromatographic peak height relative to baseline noise under the final analytical conditions.

Statistical analysis was performed using one-tailed paired Wilcoxon signed-rank tests (licensed vs. unlicensed; alternative hypothesis: licensed > unlicensed; exact method) for each panel analyte. The rank-biserial correlation (r = W/[n(n + 1)/2]) was calculated as an effect size measure. Bootstrap 95% confidence intervals on mean fold-change were computed using the percentile method with 100,000 resamples. With n = 3 paired observations, the minimum achievable one-tailed *p*-value is 0.125, which is a mathematical property of the sample size rather than a reflection of effect magnitude; all results should be interpreted accordingly.

## 5. Conclusions

This study demonstrates the feasibility of an HPLC-based multi-analyte secretome characterization panel for cAD-MSCs that quantifies adenosine and kynurenine as small-molecule outputs alongside IL-10 and TGF-β as exploratory protein outputs from conditioned media. Cytokine licensing with IFN-γ and TNF-α produced directionally consistent, panel-wide increases across all four analytes in three independent donor lines, providing initial evidence that inflammatory priming can shift MSC secretory output in a measurable, multi-pathway manner detectable by a single instrument platform.

The metabolite outputs—adenosine and kynurenine—were measured with chromatographic selectivity confirmed by spiked recovery in a conditioned media matrix and represent the validated component of the panel. The protein outputs—IL-10 and TGF-β—are presented as exploratory/semi-quantitative measurements. The panel as currently implemented comprises analyte-specific HPLC methods performed on a single instrument and uses two collection timepoints and media conditions, which constrains direct cross-analyte comparison but supports an instrument-consolidated workflow.

These findings provide a foundation for expanded development. Planned next steps include: orthogonal confirmation of IL-10 and TGF-β peak identity by immunoassay; expanded donor cohort analysis (n ≥ 6) to enable inferential statistics and expected range definition; completion of full analytical validation parameters (selectivity, precision, matrix effects, recovery, system suitability); functional correlation of panel outputs with biological potency assays such as T-cell suppression; and evaluation of whether analytical timepoints and media conditions can be harmonized to support a more integrated panel workflow.

Canine IMHA provides a biologically motivated disease context for this development program; the secretory mediators quantified here align mechanistically with the therapeutic goals of immune regulation and tolerance induction in this condition.

## Figures and Tables

**Table 1:** HPLC calibration curve performance for panel analytes.

Analyte	Detection Channel (nm)	Standard Range	N Std	Slope	Intercept	R^2^
Adenosine	260	0.1–50 µM	6	2.890	37.618	0.9997
Kynurenine	360	1–100 µM	6	0.835	0.264	1.0000
IL-10	214	0.5–100 ng/mL	5	17.878	363.851	0.9939
TGF-β	214	0.5–200 ng/mL	5	95.703	6144.310	0.9983

**Table 2:** Conditioned media concentrations and licensing-responsive fold-changes across three independent cAD-MSC donor lines (mean ± SD; CV%; n = 3).

Analyte	Units	Unlicensed (Mean ± SD; CV%)	Licensed (Mean ± SD; CV%)	Fold Change (95% CI)	Sign Test †
Adenosine	µM	21.180 ± 1.733; 8.2%	49.032 ± 3.413; 7.0%	2.32 (2.19–2.59)	3/3
Kynurenine	µM	1.736 ± 0.121; 6.9%	5.298 ± 0.292; 5.5%	3.05 (2.97–3.11)	3/3
IL-10	ng/mL	0.826 ± 0.068; 8.2%	1.309 ± 0.168; 12.8%	1.58 (1.41–1.87)	3/3
TGF-β	ng/mL	1.092 ± 0.057; 5.2%	1.825 ± 0.337; 18.5%	1.67 (1.46–2.11)	3/3

† Sign test result for directional consistency across all three donor lines (*p* = 0.125 one-tailed for each analyte; n = 3 precludes significance at conventional α = 0.05). IL-10 and TGF-β measurements are exploratory/semi-quantitative; peak identity was assigned by retention-time matching to reference standards and has not been orthogonally confirmed.

**Table 3:** Individual concentrations by analyte and condition.

Analyte	Units	Donor 1	Donor 2	Donor 3	Mean ± SD	CV%
Adenosine-Unlicensed						
Adenosine	µM	20.546	23.141	19.854	21.180 ± 1.733	8.2%
Adenosine-Licensed						
Adenosine	µM	45.111	50.647	51.339	49.032 ± 3.413	7.0%
Kynurenine-Unlicensed						
Kynurenine	µM	1.848	1.752	1.608	1.736 ± 0.121	6.9%
Kynurenine-Licensed						
Kynurenine	µM	5.490	5.442	4.963	5.298 ± 0.292	5.5%
IL-10-Unlicensed						
IL-10 †	ng/mL	0.798	0.778	0.904	0.826 ± 0.068	8.2%
IL-10-Licensed						
IL-10 †	ng/mL	1.128	1.458	1.343	1.309 ± 0.168	12.8%
TGF-β-Unlicensed						
TGF-β †	ng/mL	1.072	1.156	1.047	1.092 ± 0.057	5.2%
TGF-β-Licensed						
TGF-β †	ng/mL	1.560	1.711	2.204	1.825 ± 0.337	18.5%

† IL-10 and TGF-β values are exploratory/semi-quantitative; reported concentrations are back-calculated to original conditioned media equivalents. Peak identity assigned by retention-time matching to purified reference standards; orthogonal confirmation is required.

## Data Availability

The original contributions presented in this study are included in the article. Further inquiries can be directed to the corresponding author.

## Abbreviations

The following abbreviations are used in this manuscript:

MSC	Mesenchymal Stromal/Stem Cell
HPLC	High Performance Liquid Chromatography
TNF-α	Tumor necrosis factor alpha
IFN-γ	Interferon gamma
TGF-β	Transforming growth factor beta
IL-10	Interleukin ten
TFA	Trifluoroacetic acid
TCA	Trichloroacetic acid
IDO	Indoleamine 2,3-dioxygenase
JAK/STAT	Janus Kinase—Signal Transducer and Activator of Transcription
IMHA	Immune Mediated Hemolytic Anemia
FBS	Fetal bovine serum
MWCO	Molecular weight cut-off
CV	Coefficient of variation
SD	Standard Deviation
LOQ	Limit of Quantification
